# Decreased Left Caudate Volume Is Associated with Increased Severity of Autistic-Like Symptoms in a Cohort of ADHD Patients and Their Unaffected Siblings

**DOI:** 10.1371/journal.pone.0165620

**Published:** 2016-11-02

**Authors:** Laurence O’Dwyer, Colby Tanner, Eelco V. van Dongen, Corina U. Greven, Janita Bralten, Marcel P. Zwiers, Barbara Franke, Dirk Heslenfeld, Jaap Oosterlaan, Pieter J. Hoekstra, Catharina A. Hartman, Wouter Groen, Nanda Rommelse, Jan K. Buitelaar

**Affiliations:** 1 Radboud UMC, Donders Institute for Brain, Cognition and Behaviour, Department of Cognitive Neuroscience, Nijmegen, The Netherlands; 2 Department of Ecology and Evolution, University of Lausanne, Lausanne, Switzerland; 3 King’s College London, MRC Social, Genetic and Developmental Psychiatry Centre, Institute of Psychiatry, London, United Kingdom; 4 Department of Human Genetics, Radboud UMC, Nijmegen, The Netherlands; 5 Radboud University Medical Center, Donders Institute for Brain, Cognition and Behaviour, Department of Psychiatry, Nijmegen, The Netherlands; 6 Department of Clinical Neuropsychology, VU University, Amsterdam, The Netherlands; 7 Department of Cognitive Psychology, V.U. University, Amsterdam, The Netherlands; 8 Department of Psychiatry, University Medical Center Groningen, University of Groningen, Groningen, The Netherlands; 9 Karakter Child and Adolescent Psychiatry University Center Nijmegen, Nijmegen, The Netherlands; George Washington University, UNITED STATES

## Abstract

Autism spectrum disorder (ASD) symptoms frequently occur in individuals with attention-deficit/hyperactivity disorder (ADHD). While there is evidence that both ADHD and ASD have differential structural brain correlates, knowledge of the structural brain profile of individuals with ADHD with raised ASD symptoms is limited. The presence of ASD-like symptoms was measured by the Children's Social Behavior Questionnaire (CSBQ) in a sample of typically developing controls (n = 154), participants with ADHD (n = 239), and their unaffected siblings (n = 144) between the ages of 8 and 29. Structural magnetic resonance imaging (MRI) correlates of ASD ratings were analysed by studying the relationship between ASD ratings and grey matter volumes using mixed effects models which controlled for ADHD symptom count and total brain volume. ASD ratings were significantly elevated in participants with ADHD relative to controls and unaffected siblings. For the entire group (participants with ADHD, unaffected siblings and TD controls), mixed effect models revealed that the left caudate nucleus volume was negatively correlated with ASD ratings (t = 2.83; P = 0.005). The current findings are consistent with the role of the caudate nucleus in executive function, including the selection of goals based on the evaluation of action outcomes and the use of social reward to update reward representations. There is a specific volumetric profile associated with subclinical ASD-like symptoms in participants with ADHD, unaffected siblings and controls with the caudate nucleus and globus pallidus being of critical importance in predicting the level of ASD-like symptoms in all three groups.

## Introduction

Attention-deficit/hyperactivity disorder (ADHD) and autism spectrum disorder (ASD) are both severely impairing, highly heritable neurodevelopmental disorders [1,2] [3]. ASD is characterised by impaired social and communicative skills as well as restricted and repetitive behaviours and interests, whereas ADHD is characterised by severe inattention and/or hyperactivity and impulsivity [3]. Although the core features of both disorders appear to describe quite different phenotypes, elevated levels of ASD symptoms have been reported in ADHD, while elevated ADHD symptoms have also been reported in ASD [1], [2,4–6] [7].

The presence of milder, subclinical ASD symptoms has been shown to be continuously distributed in the general population [2,8,9], although the neural substrates of this phenomenon have yet to be explored in detail. The current study investigates subclinical ASD-like symptoms in the ADHD population and their unaffected siblings. A shared aetiology between ADHD and ASD has been observed in many domains, with deficits in executive functions and motor speed being linked to familial vulnerability for both ASD and ADHD [10–12]. Abnormalities in reward processing are also common in both ADHD [13] and ASD [14], [15], while others have documented an overlap of genetic factors that relate to both disorders [4] [16]. Brain volume abnormalities are important indicators of pathophysiological processes that likely reflect disorder aetiology [17]. A number of meta-analyses have found reduced brain volume in ADHD (with ages ranging from ~10–37 years of age) [18], [19,20]. Regional volume reductions in ADHD have been localised to the globus pallidus, putamen, caudate nucleus, lentiform gyrus and cerebellum [18], [19,20] [21]. Furthermore, both increasing age and use of stimulant medication were found to be independently associated with normalisation of grey matter (GM) volume towards that of healthy controls [19]. With regard to dimensional findings, decreased frontal and temporal GM volumes have been associated with increased ratings of attention problems in children with ADHD [22].

Abnormalities of regional volume have been found in ASD. Cerebellum [23], amygdala-hippocampal complex [24–27], frontotemporal regions [25,26,28], caudate nucleus [29,30], and nucleus accumbens [31] have all been noted to have reduced volume in individuals with ASD relative to controls, while the superior temporal gyrus (STG) has been found to be significantly increased in ASD [21]. The caudate nucleus has been found to have both reduced volume in ASD relative to controls [29,30], as well as increased volumes relative to controls [17,30,32–34]. Hemispheric asymmetry also plays a role in ASD, as previous work has suggested left-hemisphere dysfunction [35,36], while other studies have pointed to predominant right hemisphere impairment [37,38]. Thus, the direction of a laterality effect has been inconsistent across the literature. Nevertheless, some studies have indicated that there is a lower degree “leftward” cortical symmetry in ASD relative to controls [39] and that the left hemisphere is under tighter genetic control than the right hemisphere [40] which may be relevant for a highly heritable disorder such as ASD. Laterality has also been used in machine learning to attempt to discriminate between ASD cases and controls [41].

Clinical symptoms have been associated with anatomic differences, for example, abnormalities in Broca's and Wernike's areas have been related to impaired language and social communication [42]. Additionally, frontotemporal regions and the amygdala have been associated with abnormalities in socio-emotional processing [33,43,44], while the frontostriatal system has been linked to repetitive and stereotyped behaviours [29,34] in individuals with ASD.

No study to date has aimed to identify the regional volumetric correlates of elevated ASD symptoms within ADHD. In previous work using the same cohort we established that ASD ratings in ADHD were predicted by the interaction between global white matter (WM) and global GM volumes, with increasing ASD ratings associated with greater GM volume [45]. We extend this work by studying the relationship between ASD ratings and regional volumetric measures of subcortical grey matter structures in the same cohort of ADHD participants, their unaffected siblings and typically developing controls. Because of the previously noted effects of laterality on ASD symptoms [41], and also in light of the inconsistencies within the literature (specifically the direction of possible laterality effects) we wanted to examine this question in the large sample size available in the current cohort. Overall, the current cohort allows for a rare opportunity to study ADHD and ASD-like symptoms in the same individual with a view to understanding the biological underpinnings of the high comorbidity of these two disorders.

Structural and functional MRI studies have found the caudate nucleus to be altered (with both increased volume and activation and decreased volume and activation relative to controls) in ASD and to be associated with dysfunctions in multiple domains related to ASD, such as repetitive and stereotyped behaviour [46], reward processing [47] and executive function [48], [49]. Based on this literature, our hypothesis was that the caudate nucleus may have a significant role [34,46], [1,50], while structures such as the cerebellum [23], amygdala-hippocampal complex [24–27], frontotemporal regions [25,26,28], [29,30], and nucleus accumbens [31], may have a contributory but potentially subsidiary role, in predicting the extent to which subclinical ASD-like symptoms are expressed in patients with ADHD but not in their unaffected siblings or controls. However, it should be borne in mind that results to date have not been consistent, with a recent longitudinal study within a similar age range to the present study demonstrating no group differences in caudate volume between an ASD and typically developing group [51], whereas a study in children indicated an increase in the growth rate of striatal structures in individuals with autism compared with control subjects with an effect that was specific to the caudate nucleus, where growth rate was doubled [52]. Nevertheless, overall a meta-analysis by Stanfield et al. [53] found that the total brain, cerebral hemispheres, cerebellum and caudate nucleus were increased in volume, whereas the corpus callosum area was reduced. A subsequent meta-analysis [54] closely matched these findings suggesting a convergence between volumetric and VBM data that adds support to the potential role of the caudate nucleus in the pathophysiology of autism.

Previous research has also indicated that frontostriatal areas in general play an important role in modulating reward and motivation which in turn influence the expression of ASD symptom in ADHD [55], [47]. Subcortical brain volumes were segmented to investigate the role of the caudate, while also allowing for the investigation of other structures that have been less frequently implicated in ASD symptoms, such as the globus pallidus, the nucleus accumbens, putamen [26], thalamus [56], brain stem [57], hippocampus, and amygdala [58].

## Methods

### Participants

Participants were selected from a follow-up (2009–2012) of the Dutch part of the International Multicenter ADHD Genetics (IMAGE) study, performed between 2003–2006 (as described in detail in [59–62]. Written informed consent forms were obtained for all participants. MRI was part of the NeuroIMAGE protocol, while no MRI was performed for the original IMAGE sample. Next of kin (parents) signed written informed consent forms for participants under 12 years of age. For children 12–18 years of age, next of kin (parents) as well as participants themselves signed written informed consent forms. The study was approved by the local medical ethical committee (Centrale Commissie Mensgebonden Onderzoek Regio Arnhem-Nijmegen and the ethics committee of the VU Medical Center in Amsterdam).

At first enrolment for IMAGE, 365 families with at least one child with combined type ADHD and at least one biological sibling (regardless of ADHD diagnosis) were recruited, in addition to 148 control families with at least one child, with no formal or suspected ADHD diagnosis in any of the first-degree family members. Recruitment of ADHD families was accomplished through probands with ADHD attending outpatient clinics in the regions Amsterdam, Groningen, and Nijmegen, as well as a Vrije Universiteit Amsterdam (VU University) affiliated ADHD research institute. Control families were recruited through primary and high schools in the same geographical regions as the participating ADHD families. All family members, also those who did not participate in IMAGE, were invited for follow-up measurement with a mean follow-up period of 5.9 years (*SD* = .72) in the NeuroIMAGE study (www.neuroimage.nl). In order to balance the distribution of gender and age between the ADHD and healthy control groups, additional girls with ADHD (any type; N = 50) and healthy control boys (N = 50) were recruited for NeuroIMAGE. Inclusion criteria were the same for all participants, and largely consistent with IMAGE: participants had to be between 5–30 years, of European Caucasian descent, have an IQ ≥ 70 and no diagnosis of autism, epilepsy, general learning difficulties, brain disorders and known genetic disorders (such as Fragile X syndrome or Down syndrome). Relating to the NeuroIMAGE MRI protocol, participants were excluded if they were younger than 8 years or had any contra indication to MRI scanning (e.g. implanted metal or medical devices, or possible pregnancy). 79% of participants from the IMAGE study also participated in the NeuroIMAGE follow-up study with no evidence for selective attrition [63]. Combined with newly recruited participants the NeuroIMAGE study tested a total of 1085 participants.

For the current study, participants were selected from the total data set when the following data was available: a high quality T1 weighted MPRAGE image, complete information from the Children’s Social Behavior Questionnaire (providing information on the autism spectrum symptoms) [64], complete information from the Schedule for Affective Disorders and Schizophrenia for School-Age Children—Present and Lifetime Version (K-SADS-PL) and the Conners ADHD questionnaire. IQ information and medication history were also required in order to include participants in the current study. Participants with a subthreshold ADHD diagnosis were excluded. A subthreshold ADHD subject is defined as a subject from a family with a known ADHD history and at least one affected sibling during recruitment, and must also have between 2 and 5 ADHD symptoms according to the Conners/K-SADS screen. A full description of the NeuroIMAGE study design is a paper by von Rhein et al. [63].

### ADHD Diagnostic Assessment

To determine ADHD diagnoses at the follow-up measurement, all participants in the study were assessed using a combination of Conners' ADHD questionnaires [65–67] and a semi-structured diagnostic interview. For participants using medication, ratings were obtained for children’s functioning when off medication. A full description of the diagnostic algorithm is provided in the Supplementary Information and also in a NeuroIMAGE study design paper [63].

### ASD measures

The parent-reported Children’s Social and Behavior Questionnaire (CSBQ) contains 49 items on a 3-point Likert scale. All items in the CSBQ are found in S2 Text. It contains items that refer directly to the DSM-IV criteria for autism, but it also captures more subtle symptoms of ASD. Therefore, it is suitable for measuring behavioural problems in children with milder variants of ASD. CSBQ items are grouped into the following six subscales: (1) “Tuned” (tuning emotions and behaviour to the current situation), (2) “Social interest” (social interest, motivation and reciprocity), (3) “Orientation” (orientation in space and time), (4) “Social understanding” (ability to understand social context), (5) “Resistance” (fear and resistance to change) and (6) “Stereotypy” (repetitive motor and sensory behaviour and stereotypy). The CSBQ has good internal, test-retest and inter-rater reliability, and demonstrated convergent and divergent validity [64]. Additionally, to assess the content validity of the CSBQ, it has previously been compared to an autism screening instrument, the Autism Behavior Checklist (ABC) [68]. A strong correlation of 0.75 was found between the total scores of both questionnaires in a large Dutch population sample [69]. The CSBQ has also been compared with the Autism Diagnostic Interview-Revised (ADI-R), Autism Diagnostic Observation Schedule (ADOS) and clinical classification in children with mild and moderate intellectual disability. High coherence with all three classification methods were reported [70].

Similar to previous studies [71,72], an aggregate score from four subscales, (1) Social interest, (2) Social understanding, (3) Stereotypy and (4) Resistance, was used to capture the core ASD-like symptoms. The remaining two CSBQ subscales (Tuned and Orientation) probe dysfunctional social behaviours which, although characteristic for ASD, are also related to the ADHD dimensions of hyperactivity/impulsivity and attention problems respectively [64]. In order to specifically focus on ASD these subscales were not considered in the current study. We prospectively planned to use four subscales to disentangle ASD and ADHD correlates. The current approach ensures that a high score means that a participant has a substantial amount of symptoms that can be definitely characterized as ASD-like, as defined in the DSM. Without this approach there would be a risk of obtaining a high ASD score that in reality would be a (hidden) high ADHD score.

### Procedure

During the testing day, participants were motivated with short breaks, and at the end of the day, children received a reward of €50 and a copy of their anatomical MRI scan. Informed consent was signed by all participants (parents signed informed consent for participants under 12 years of age), and the study was approved by the local medical ethical committee (Centrale Commissie Mensgebonden Onderzoek Regio Arnhem-Nijmegen and the ethics committee of the VU Medical Center in Amsterdam).

#### High Resolution T1 Structural Image Acquisition and Processing

Whole brain T1 weighted MPRAGE images were acquired at 1.5T using an 8 channel phased array headcoil on a Siemens Sonata scanner at the VU University in Amsterdam and a Siemens Avanto MR scanner at the Donders Institute for Brain, Cognition and Behaviour in Nijmegen. A breakdown of the distribution of subjects scanned at the two sites is included in S1 Table. Sequence parameters were as follows: TI/TE/TR = 1000/2.95/2730 ms, imaging matrix 256 x 256, 176 slices, voxel size 1 x 1 x 1 mm^3^, GRAPPA acceleration 2.

#### FIRST Structural Image Processing

The FIRST algorithm was applied to separately estimate the left and right volumes of eight regions; amygdala, hippocampus, nucleus accumbens, caudate nucleus, putamen, pallidum, thalamus and brain stem. FIRST is part of FMRIB's Software Library (FSL) and performs both registration and segmentation [73]. Within the FIRST software, the Dice overlap measures (similarity coefficient) [74] ensures that for structures with large surface-area-to-volume ratios, such as the caudate, small differences in surface error are heavily penalized, since an average error of one voxel at the boundary will substantially affect the volume overlap [73].

During registration, the input data (3D T1 images) were transformed to the MNI (Montreal Neurological Institute) 152 standard space, by means of affine transformations based on 12 degrees of freedom [75–77]. This registration allowed intracranial volume (ICV) to be estimated by scaling the volume of the MNI 152 brain with the determinant of the subject's inverse affine transformation matrix, also known as the Atlas Scaling Factor [78].

After subcortical registration, a sub-cortical mask was applied, to locate the different subcortical structures, followed by segmentation based on shape models and voxel intensities. Absolute volumes of structures were calculated, taking into account the transformations made in the first stage [73]. The following formula was used to compute normalised volumes of each deep grey matter structure:

$$\text{total GM volume of structure }\left({\text{mm}}^{\text{3}}\right)\text{ / ICV}$$

An average of two T1 scans was used for volume calculation In most instances, an average of two T1 scans, that were acquired on the same testing day, were used for volume calculation (n = 509). For 28 individuals only one T1 scan was available for volume calculation.

#### Statistics

Our approach was to begin by developing an initial behavioural factors or “external” model to determine which features, other than brain regions (i.e. “external” to the brain), would affect ASD ratings, calculated as the log-transformed aggregate of the four CSBQ subscales ('Social interest', 'Social understanding', 'Stereotypy' and 'Resistance'). We used a mixed-effects general linear model [79] with age, gender, diagnosis, MRI site, current medication status, IQ and their interactions with each other as potential fixed factors in the full external model. Diagnosis was coded with three levels; Control, Unaffected Sibling and ADHD. MRI site was coded with two levels for Amsterdam or Nijmegen. Site effects were previously tested for in a NeuroIMAGE design paper that examined many aspects of the entire NeuroIMAGE cohort [63]. This design paper studied the grey matter volumes (GMV) for the two sites and indicated that there is no significant site effect within the NeuroIMAGE cohort. Current medication status was also coded with two levels for currently on medication, or currently not on medication. Because of the repeated measures within families inherent in our sampling protocol and the known influence of ADHD symptoms on the variance in ASD ratings [80,81], [61], [7], family (i.e. the unique family ID, with siblings from the same family having the same ID) and total ADHD symptom count (which was calculated according to the algorithm described in detail in S1 Text) were included as random effects in the analysis. By removing effects in a stepwise manner, and assessing model fit using analysis of deviance tests on nested models [79], we simplified the initial model. The final “external” model included significant non-brain factors (age, gender and diagnosis) and all significant interactions.

Next, we developed an initial brain volumes model to determine which of the subcortical brain regions under investigation significantly affected ASD ratings. We termed this an “internal” model as it contained only brain structures. Because of the previously found effects of laterality on ASD symptoms [41], we separated brain regions, except for the brain stem, into right and left hemispheres and analysed the two resulting models separately using ridge regression [82,83]. Our intention was to investigate right and left structures separately, with the hypothesis based on the above cited literature that the two hemispheres would differ with respect to the response variable of the model, which is ASD rating. A direct comparison of the right and left hemispheres is useful to shed more light on this area.

Ridge regression is a robust way of dealing with the problem of variance inflation and parameter mis-estimation associated with correlated explanatory variables [84], as were brain region volumes in our study (right-sided correlations: 0.57 ≤r ≤0.94; left-sided correlations: 0.58 ≤ r ≤ 0.92). We followed the same removal/addition stepwise approach as with the external model to develop final left and final right internal models. To test if one model was better than the other at predicting the data, we compared the final comprehensive right and comprehensive left models using an analysis of deviance test [79]. We then included the results of these two final internal models into the final external model, producing final comprehensive left and final comprehensive right models. This merging of the external and internal models, simply involves taking the significant factors from the right internal model and including these in the external model to produce a final comprehensive right hemisphere model. Similarly, the left hemisphere model involves taking the significant factors from the left internal model and including these in the external model to produce a final comprehensive left hemisphere model.

For the left hemisphere ‘internal’ models, the globus pallidus and caudate nucleus were returned as significant. When including the left globus pallidus and left caudate nucleus into the external model to create the final comprehensive left hemisphere model, we followed Graham's [84] sequential regression method for dealing with the resulting collinearity (caudate nucleus-globus pallidus: r = 0.881, *P* < 0.001). We removed this collinearity by using the residuals of caudate nucleus regressed on globus pallidus values. We also included the potential for interaction between brain regions in affecting ASD rating.

For the right hemisphere ‘internal’ models, ridge analysis did not reveal any significant brain structures, and thus the final comprehensive right hemisphere model collapsed to the generic ‘external’ model.

As a complementary test to assess whether one side of the brain would better explain ASD ratings, we fit separate linear models for each hemisphere (right and left) of each brain structure on the log-transformed ASD spectrum scores. We then directly compared the respective fits of right and left hemispheres for each structure using separate Cox likelihood ratio tests (with the lmtest package in R) for non-nested model comparisons [85,86]. For each test, two comparisons were made: one for the significant improvement of right-sided models when left-sided data were included, and the other for the improvement of left-sided models when right-sided data were included. Because the likelihood ratio tests were not independent (i.e. they tested the results of models on structures that were themselves correlated), we controlled for the inflated false-discovery rate by adjusting their resulting p values according to the Benjamini-Hochberg correction for non-independent tests [87]. We then used these corrected p values in a Fisher's omnibus meta-analysis [88] to determine which side of the brain overall better explains ASD ratings.

## Results

### Demographic and Cognitive Characteristics

The demographic characteristics of the cohort are shown in Table 1.

**Table 1:** Demographic Table.

	Con		Unaffected Sibling		ADHD						
N	154		144		239						
	Mean	SD	Mean	SD	Mean	SD	F	p	US—Con	ADHD—Con	ADHD—US
Age	17.12	3.44	17.19	3.9	17.3	3.2	F(2,441) = 0.108	0.897	0.986	0.893	0.961
IQ	107.51	13.63	101.58	13.85	97.8	15.2	F(2,441) = 21.2	<0.0001	0.00122	<0.0001	0.0354
Social interest	0.91	1.72	1.88	3.33	4.5	4.5	F(2,441) = 54	<0.0001	0.0528	<0.0001	<0.0001
Social understanding	1.02	1.69	1.35	1.58	5.1	3.7	F(2,441) = 140	<0.0001	0.54	<0.0001	<0.0001
Stereotypy	0.3	0.75	0.33	0.95	2	2.5	F(2,441) = 59	<0.0001	0.984	<0.0001	<0.0001
Resistance	0.35	0.81	0.46	0.92	1.4	1.7	F(2,441) = 38.9	<0.0001	0.765	<0.0001	<0.0001
ASD total	2.58	3.7	4.03	4.82	13.1	9.5	F(2,441) = 129	<0.0001	0.181	<0.0001	<0.0001
ADHD total	0.45	0.98	0.75	1.42	13.5	2.9	F(2,441) = 2420	<0.0001	0.442	<0.0001	<0.0001
Gender	92/62		87/57		75/164			<0.0001			

Values are mean ± standard deviation. Significance was set at p < 0.05. All p values refer to ANOVAs, except for gender where the p value refers to a chi-square test. Where ANOVA's returned a significant result, post-hoc Tukey Honest Significant Difference (Tukey HSD) tests were performed. US—Con, refers to a pairwise comparison between unaffected siblings and controls. ADHD-Con, refers to a a pairwise comparison between ADHD and controls. ADHD—US, refers to a pairwise comparison between ADHD and unaffected siblings. Four subscales of the Children's Social and Behavioural Questionnaire (CSBQ) which probe ASD spectrum symptoms are shown in this table: Social Interest, Social Understanding (Understanding), Stereotypy and Resistance. ASD-total is calculated as a sum of these four subscales. ADHD-total scores are calculated according to the algorithm described in detail in S1 Text. For gender, females are noted first, with the format female number / male number.

### Mixed-effects models for ASD-like symptoms modelled against subcortical volumes

**Fig 1:**
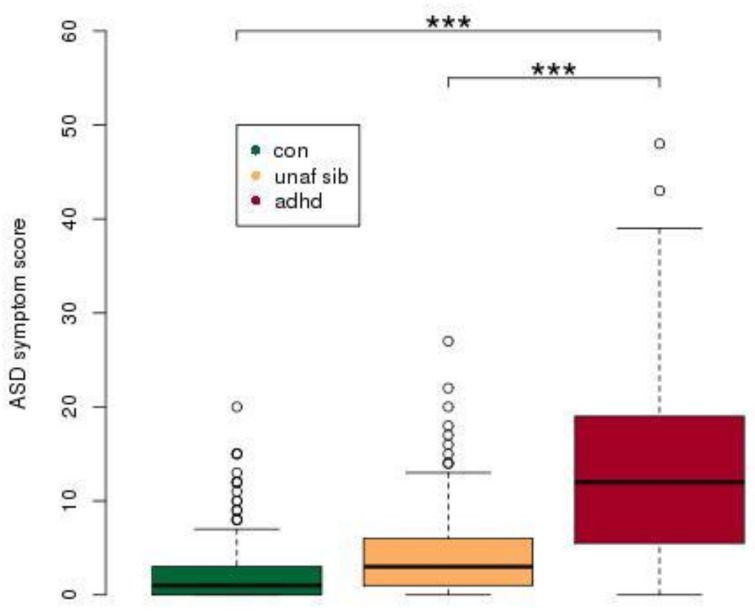
ASD-like symptoms in healthy controls, unaffected siblings and ADHD. Participants with ADHD were found to have significantly higher scores relative to both unaffected siblings and healthy controls. *** p<0.001, with post-hoc Tukey test, following an ANOVA. ASD symptom score refers to an aggregate score from the four Children's Social and Behavioural Questionnaire (CSBQ) subscales, (1) Social interest, (2) Social understanding, (3) Stereotypy and (4) Resistance. Abbreviations: con, control; unaf sib, unaffected siblings, adhd, Attention-Deficit/Hyperactivity Disorder.

The final external model of ASD ratings included the following effects: age, gender, diagnosis and an age by diagnosis interaction. The ADHD group had higher overall ASD scores than did the control group (*t* = 5.61, *P* < 0.001), but ASD scores of unaffected siblings and typically developing controls did not differ (*t* = 1.68, *P* = 0.093) (Fig 1).

**Fig 2:**
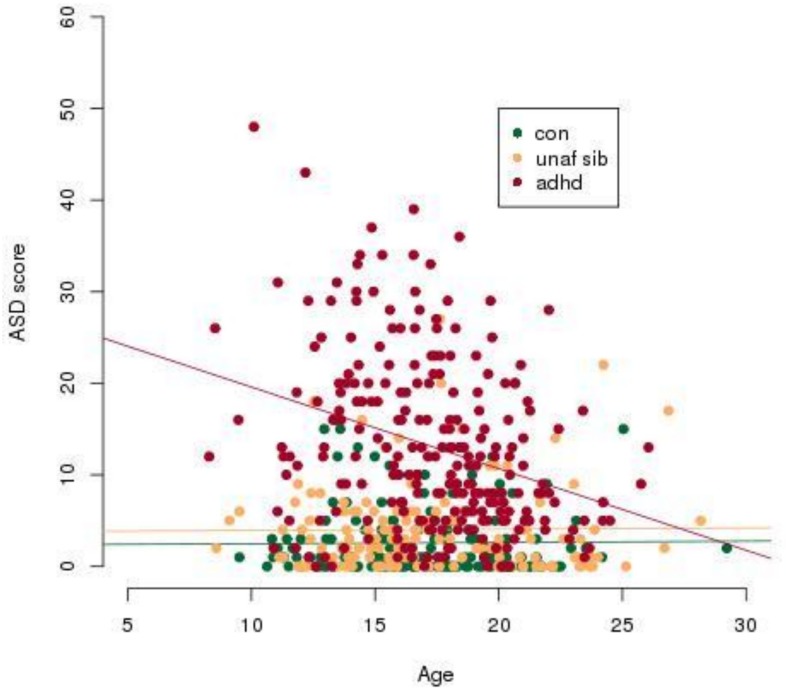
ASD scores decrease significantly with age. Only the regression line for ADHD participants returned a significant Pearson's product-moment correlation with p<0.05. ASD score refers to an aggregate score from the four Children's Social and Behavioural Questionnaire (CSBQ) subscales, (1) Social interest, (2) Social understanding, (3) Stereotypy and (4) Resistance. Abbreviations: con, control; unaf sib, unaffected siblings, ADHD, Attention-Deficit/Hyperactivity Disorder; ASD, Autism Spectrum Disorder.

**Fig 3:**
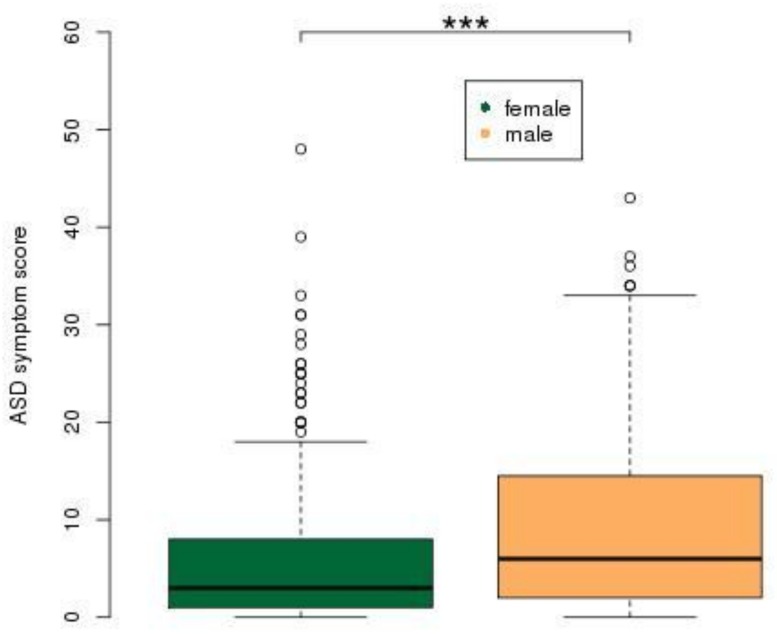
ASD scores significantly increased in males. ASD score refers to an aggregate score from the four Children's Social and Behavioural Questionnaire (CSBQ) subscales, (1) Social interest, (2) Social understanding, (3) Stereotypy and (4) Resistance. Abbreviations: con, control; unaf sib, unaffected siblings, ADHD, Attention-Deficit/Hyperactivity Disorder; ASD, Autism Spectrum Disorder.

ASD ratings for the entire group showed an insignificant decline with age (*t* = 0.036, *P* = 0.971), which was entirely due to the ADHD diagnosis group (Fig 2). Although the ADHD group differed (*t* = -2.51, *P* = 0.013) from the control group with respect to the effect of age, the unaffected sibling and control groups did not differ with respect to the effect of age (*t* = -0.979, *P* = 0.328; Fig 2). Finally, males had higher ASD ratings than did females (*t* = 2.09, *P* = 0.037; Fig 3).

**Fig 4:**
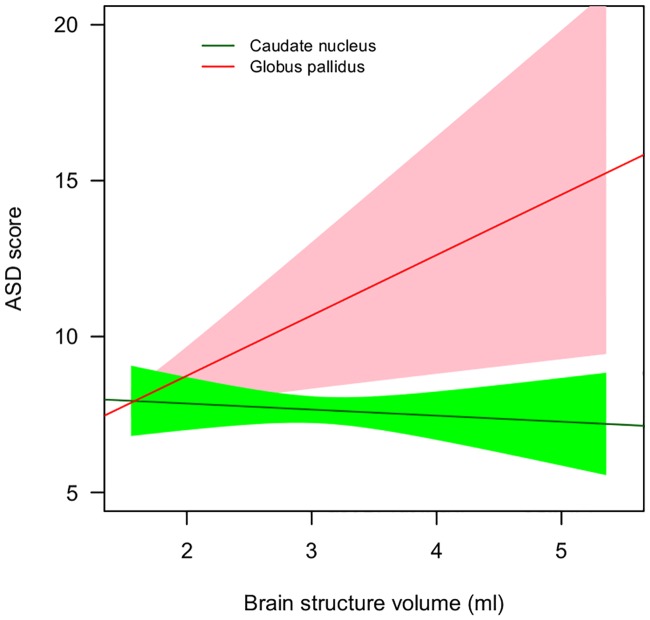
ASD scores are predicted by an interaction between left caudate nucleus volume and left globus pallidus volume. Confidence intervals of 75% are shown for both structures. ASD symptom score refers to an aggregate score from the four Children's Social and Behavioural Questionnaire (CSBQ) subscales, (1) Social interest, (2) Social understanding, (3) Stereotypy and (4) Resistance. Abbreviations: con, control; unaf sib, unaffected siblings, adhd, Attention-Deficit/Hyperactivity Disorder; ASD, Autism Spectrum Disorder.

For the internal models of the effect of brain region on ASD rating, the final left-sided model included the volumes of the caudate nucleus (scaled *t* = 2.83; *P* = 0.005) and the globus pallidus (scaled *t* = 3.21, *P* = 0.001; Fig 4). No brain structures remained significant in the final right-sided internal model. The final, comprehensive left-sided model (Table 2) included subject age, gender, diagnosis, age by diagnosis interaction, caudate nucleus volume, globus pallidus volume and caudate nucleus by globus pallidus interaction. When plotted, the interaction between caudate nucleus and globus pallidus described a situation where low ASD ratings were accompanied by low caudate nucleus volume coupled with high globus pallidus volume, whereas high ASD ratings were accompanied by high caudate nucleus volume coupled with low globus pallidus volume (Fig 4). In comparing the final left- and right-sided comprehensive models, the model including left brain structures explained the data significantly better than did the external model (AIC left model = 1317.1 versus AIC right model = 1324.2, *P* = 0.006), which was the same as the final right-sided comprehensive model (since none of the subcortical structures was significantly related to the ASD score).

**Table 2:** Final, left-sided comprehensive generalised linear mixed-effect model for ASD.

Effect	*t*	*P*
Age	-0.404	0.687
Gender	2.75	0.006
Diagnosis (unaffected sibling vs. control)	1.34	0.180
Diagnosis (ADHD vs. control)	5.06	<0.001
Left caudate nucleus (residuals)	-2.13	0.034
Left globus pallidus (residuals)	-1.45	0.147
Age:diagnosis (unaffected sibling vs. control)	-0.704	0.482
Age:diagnosis (ADHD vs. control)	-2.09	0.037
Caudate nucleus (residuals):Globus pallidus (residuals)	-1.76	0.079*

Caudate nucleus and globus pallidus values are regression residuals (see results section).

* Although the caudate nucleus:globus pallidus interaction was only marginally significant, removing this interaction significantly reduced the model fit (*P* = 0.034). A general mixed-effect model is run using normalised volumes of the left nucleus accumbens, age, diagnosis and gender as explanatory variables together with family and total ADHD symptoms as random effects. The log-transformed ASD score is set as the response variable. The final model is derived in an iterative model selection procedure by removing insignificant effects in a stepwise manner, and checking for model fit using analysis of deviance tests on nested models. (See Methods for detailed description of model selection procedure). ASD score refers to an aggregate score from the four Children's Social and Behavioural Questionnaire (CSBQ) subscales, (1) Social interest, (2) Social understanding, (3) Stereotypy and (4) Resistance. Abbreviations: df, degrees of freedom

In addition to the left-sided final comprehensive model explaining the data better than the right-sided final model, we investigated whether or not adding left structures to the final right ‘internal’ model improved the data fit, or vice-versa if adding right structures to the final left ‘internal’ model improved the data fit. Individual right-sided models for the hippocampus, amygdala and globus pallidus were significantly improved by including data from their left-sided counterparts in the models (Fisher's omnibus meta-analysis; right-sided model improved by including left side: χ^2^ = 57.88, p < 0.001; left-sided model improved by including right side: χ^2^ = 9.33, p = 0.809) (Table 3). On the other hand, no left-sided models were significantly improved by including right-sided structures.

**Table 3:** The effect of laterality on ASD spectrum scores.

Structure	Right-sided improved by left		Left-sided improved by right	
	z	p_adj_	z	p_adj_
Nucleus Accumbens	-0.351	0.764	-1.41	0.556
Hippocampus	-9.06	<0.001	0.564	0.573
Amygdala	-13.04	<0.001	0.647	0.573
Putamen	-0.943	0.605	0.584	0.573
Caudate Nucleus	0.300	0.764	-0.651	0.573
Globus Pallidus	-7.34	<0.001	2.06	0.275
Striatum	0.519	0.764	-0.726	0.573

To test for an effect of laterality of brain region on ASD spectrum scores, we fit separate linear models for each structure and hemisphere on the log-transformed ASD spectrum scores. The respective fits of right and left hemispheres for each structure were then directly compared using separate likelihood ratio tests for non-nested model comparisons using the lmtest package in R. For each test, two comparisons are made: one for the significant improvement of right-sided models when left-sided data are included, and the other for the improvement of left-sided models when right-sided data are included. Because the likelihood ratio tests were not independent (i.e. they tested the results of models on structures that were themselves correlated), we controlled for the inflated false-discovery rate by adjusting their resulting p-values according to Benjamini and Hochberg's approach for non-independent tests (See Methods for detailed description).

## Discussion

The primary finding from the current study is that an interaction between left caudate nucleus and left globus pallidus was predictive of ASD-like symptoms as measured by the CSBQ. High ASD ratings were accompanied by an increase in left caudate nucleus volume coupled with decreased left globus pallidus volume, whereas low ASD ratings were accompanied by lower left caudate nucleus volume coupled with increased left globus pallidus volume. ASD-like symptoms were also found to be significantly elevated in participants with ADHD relative to both unaffected siblings and controls. This agrees with previous studies that have found elevated levels of ASD symptoms in participants with ADHD [7,61,80,81]. Overall, left hemisphere data was found to provide a much better fit of data, which is consistent with work that has found that the left hemisphere is more abnormal in ASD [39,40].

The results indicate that the caudate nucleus and the interaction between the caudate nucleus and the globus pallidus can predict part of the variance in ASD ratings in participants with ADHD as well as in unaffected siblings and controls. Frontostriatal circuits have been implicated in many functions relating to reward and motivation as well as psychiatric disorders including ADHD and ASD [47]. Within the striatum itself, the caudate nucleus guides the selection of goals based on the evaluation of action outcomes, while the dorsal striatum updates the reward value of chosen actions which in turn influences future behaviour [89]. Aberrant development of caudate nucleus and globus pallidus may lead to diminished motivation to attend to social stimuli, such as facial expressions and voices. Indeed, a recent study found a striking pattern of under-connectivity in resting state functional MRI data between left hemisphere voice-selective posterior superior temporal sulcus (pSTS) and distributed nodes of the reward pathway in autism [15]. Therefore attending to voices may not be inherently rewarding for those with ASD [90]. The interaction between globus pallidus and caudate nucleus may be related to a bottleneck of information flow between the caudate as input areas of the striatum and globus pallidus as output area [47]. It is possible that dysfunction, originating in the caudate, leads to a compensatory offset in the globus pallidus. However, the reverse scenario is also possible, with dysfunction in the globus pallidus causing a compensatory offset in the caudate nucleus. Further analysis, particularly with tractography would be needed to examine this hypothesis. Feasibility of detailed tractography within the striatum has recently been demonstrated [91,92]. It should be borne in mind that our finding is specific for autism-like symptoms as the volumes of the caudate and globus pallidus did not correlate with ADHD symptoms.

The caudate nucleus is also integral to executive function, and has been implicated in the development of stereotyped and repetitive behaviours [93]. A number of studies have found increases in caudate volume in ASD [17,30,32–34]. Moreover, the striatum has been implicated in repetitive behavior across neuropsychiatric disorders, from Tourette’s disorder to obsessive-compulsive disorder [30,32,33,46,94–96].

Studies using a dimensional assessment of ASD symptoms, when investigating the relationship between caudate volume and ASD, have produced equivocal results. For instance, the current scientific literature shows no clear direction in the relationship between repetitive behavior and caudate volume in ASD. One of the largest studies to date (with 99 ASD participants and 89 TD participants) [46] noted a negative correlation between caudate volume and high-order repetitive behaviour. This study also found a larger caudate volume in ASD compared to controls. This counter-intuitive result may reflect a compensatory mechanism, with a larger caudate volume enabling a degree of adaptation that counters stereotyped behaviours.

Contrary to the above finding, a study with a small number of subjects (n = 12) found a positive correlation between high-order repetitive behaviour and caudate volume [32]. Further complicating a clear interpretation, a study measuring a combination of high and low-order repetitive behaviour also found a positive correlation between this measure and caudate volume [33]. Despite these discrepancies, altogether these results indicate that the caudate has a significant role in predicting more severe repetitive behaviours [32,33,46]. We should also point out that inconsistent findings in previous volumetric studies of ADHD and ASD may be related to issues of comorbidity that have generally been ignored to date.

The fact that only the left caudate predicts ASD-like symptoms is suggestive of a lateralized dysfunction in ASD, something that has been reported previously [97]. In addition, data from the left hemisphere significantly improved the fit of right hemisphere models, which is consistent with previous work that has found that the left hemisphere is more abnormal in ASD [39,40].

In our statistical model, the globus pallidus was not a significant factor by itself, which is largely consistent with previous studies [98,99]. The interaction between caudate nucleus and globus pallidus is particularly interesting as the caudate nucleus functions as an input area for the striatum while the globus pallidus functions as an output area. All parts of the cerebral cortex give rise to efferent fibers to the caudate. As an outflow of the striatum, the globus pallidus projects to the ventral nuclei of the thalamus. The interaction between the caudate nucleus and globus pallidus suggests that the inflow and outflow of the striatum is disturbed in autism.

The absence of a significant interaction between structural volume and diagnosis indicates that the caudate and globus pallidus volumes predict ASD ratings equally for typically developing controls and unaffected siblings as for ADHD participants. This was contrary to our expectation and leads to the conclusion that dysfunction in the anatomy of the striatum may influence the degree of ASD-like symptoms independent of clinical ADHD or ASD pathology.

The hypothesis that these structures would be significant in our models in controls and unaffected siblings, as well as in patients with ADHD, stems from the fact that elevated levels of ASD at a subclinical level have been reported extensively (2,5–7,48), while subclinical levels of ASD have also been shown to be continuously distributed in the general population [2,8,9].

The higher ASD ratings noted in male subjects are consistent with previous studies [100], and also reflect the fact that the male:female ratio for clinical ASD can be as high as 4 to 1 [101]. ADHD itself is also more prevalent in males [102]. The current results indicate that ASD-like symptoms are raised in males regardless of diagnosis, with no gender by diagnosis interaction. This ties in with the absence of a structure by diagnosis interaction mentioned previously, suggesting that the current profile of raised ASD ratings in males, in conjunction with raised caudate nucleus volume and lower globus pallidus volume, is applicable to the population as a whole and not only to those with a diagnosis of ADHD.

There was a significant age-related decrease in ASD-like symptoms in the participants with ADHD, which is consistent with previous cross-sectional and longitudinal studies finding age-related improvements in ASD [103,104] [105]. Normal development is associated with marked changes in myelination in WM tracts, with myelination increasing throughout childhood and adolescence [106]. Increasing myelination of frontostriatal connections during adolescence and early adulthood may facilitate top-down improvements in executive control [107]. These improvements may be related to cognitive and behavioural changes, including social adaptation, use of cognitive therapy or pharmaceutical treatment in ADHD participants over the course of adolescence.

The current study should be viewed in the context of some strengths and limitations. Clear strengths of the work are the large sample size, the inclusion of unaffected siblings of ADHD participants, as well as the use of the CSBQ to probe subclinical ASD symptoms in ADHD participants–an area of study that has been significantly neglected. Limitations include the cross-sectional design of the study. Future longitudinal studies of MRI-measured developmental trajectories are needed for assessing the impact of age on developing brain structures.

Overall, the current results highlight a specific volumetric profile that is associated with subclinical ASD symptoms in participants with ADHD, unaffected siblings and controls. The results point to the caudate nucleus and globus pallidus volumes as being of critical importance in predicting the level of ASD-like symptoms of participants with ADHD indicating that an interaction between these two structures was a significant predictor of ASD scores.

## Supporting Information

S1 Text The diagnostic algorithm for ADHD in the NeuroIMAGE sample. (DOCX)

S2 Text A description of the Children's Social and Behavioural Questionnaire (CSBQ). (DOCX)

S1 Table Distribution of Scanning over Two Sites. (DOCX)

S2 Table Full data used for all Calculations. (XLS)
