# Supplementary material for: Decreased Left Caudate Volume Is Associated with Increased Severity of Autistic-Like Symptoms in a Cohort of ADHD Patients and Their Unaffected Siblings
Source: PLoS One. 2016 Nov 2;11(11):e0165620. doi: 10.1371/journal.pone.0165620 (PMC5091763; doi:10.1371/journal.pone.0165620)
Supplement: S1 Text — (DOCX) [file pone.0165620.s001.docx]

**S1 Diagnostic Algorithm**

*Diagnostic algorithm for ADHD in the NeuroIMAGE sample*

Participants were administered the Dutch translation of the Schedule for Affective Disorders and Schizophrenia for School-Age Children - Present and Lifetime Version (K-SADS-PL): Kaufman et al., 1997) which is compatible with the DSM-IV-TR (American Psychiatric Association (116)) , Diagnostic and Statistical Manual of mental disorders, Text Revision [DSM-IV-TR] 2000). Both parents and children, if ≥ 12 years old, were interviewed separately and were initially only administered the ADHD screening interview. Participants with elevated screen scores were administered the full ADHD section. For participants using medication, ratings concerned functioning off medication. Furthermore, each child was assessed with a parent-rated questionnaire (Conners’ Parent Rating Scale – Revised: Long version (CPRS-R:L) combined with either a teacher-rating (Conners’ Teacher Rating Scale – Revised: Long version (CTRS-R:L) applied for children <18 years or a self-report Conners’ Adult ADHD Rating Scales – Self-Report: Long version (CAARS-S:L) applied for children ≥18 years. A diagnostic algorithm was applied to combine symptom counts on the K-SADS-PL and CTRS-R:L (for participants <18) or CAARS-S:L (for participants ≥18), both providing operational definitions of the 18 behavioural symptoms defined by the DSM-IV-TR. Symptoms of the questionnaire were only used in the algorithm if at least 2 symptoms were reported on the questionnaire (based on three scales: DSM Inattentive, DSM Hyperactive-Impulsive and DSM Total). Participants with a combined symptom count of ≥6 symptoms of hyperactive/impulsive behaviour and/or inattentive behaviour were diagnosed with ADHD, provided they a) met the DSM-IV criteria for pervasiveness and impact of the disorder, b) had an age of onset before 12, and c) received a *T*≥63 on at least one DSM ADHD scale of the ADHD questionnaire. Unaffected participants were required to receive a *T* <63 on all scales of the ADHD questionnaire and have ≤3 symptoms derived from the combined symptom counts of the K-SADS-PL and CTRS-R:L/CAARS-S:L. For participants ≥18 years a combined symptom count of 5 symptoms was sufficient for a diagnosis and with ≤2 symptoms they were considered unaffected. Participants not meeting criteria for ADHD or unaffected status were labelled 'subthreshold ADHD'. Diagnostic procedure for parents were similar to those applied for children ≥18 years. The ADHD questionnaire was completed by their partner (Conners' Adult ADHD Questionnaire - Observer: Short version (CAARS-O:SV). A retrospective childhood diagnosis was established in addition to a current diagnosis, using the same diagnostic algorithm used for young adults. Parents with a current and/or childhood diagnosis were labelled as affected (*n* = 7 having only a childhood diagnosis ADHD).
