# Supplementary material for: Decreased Left Caudate Volume Is Associated with Increased Severity of Autistic-Like Symptoms in a Cohort of ADHD Patients and Their Unaffected Siblings
Source: PLoS One. 2016 Nov 2;11(11):e0165620. doi: 10.1371/journal.pone.0165620 (PMC5091763; doi:10.1371/journal.pone.0165620)
Supplement: S2 Text — (DOCX) [file pone.0165620.s002.docx]

*Children's Social and Behavioural Questionnaire (CSBQ) Summary*

| ***Tuned*** | ***Social Interest*** |
| --- | --- |
| 1. Gets angry quickly.  2. Does not know when to stop.  3. Extremely stubborn.  4. Stays angry for a long time, e.g. when he/she does not get his/her way.  5. Is disobedient.  6. Draws excessive attention to him/herself.  7. Shows sudden changes of mood.  8. Makes a fuss over little things; ‘‘makes a mountain of a mole-hill’’.  9. Over-reacts to everything and everyone.  10. Cannot be corrected in situations in which he/she has done something wrong.  11. Makes inconsiderate remarks, e.g. remarks that are painful to others. | 1. Has little or no need for contact with others.  2. Makes little eye contact.  3. Does not seek comfort when he/she is hurt or upset.  4. Dislikes physical contact, e.g. does not want to be touched or hugged.  5. Does not respond to initiatives by others, e.g. does not play along when asked.  6. Does not initiate play with other children.  7. Acts as if others are not there.  8. Lives in a world of his/her own.  9. Does not display his/her feelings using facial expressions and/or bodily posture.  10. Does not look up when spoken to.  11. Cannot be made enthusiastic about anything.  12. Show no empathy when someone else is hurt or sad. |
| ***Orientation*** | ***Social Understanding*** |
| 1. Does things without realizing their aim, e.g. has to be constantly reminded to finish things.  2. Acts without consideration for the proper timeline of an activity (beginning, middle, and end).  3. Has no sense of time.  4. Has trouble taking in information  5. Has trouble doing 2 simple things simultaneously.  6. Does not appreciate danger.  7. Gets lost easily.  8. Barely distinguishes between strangers and familiar people. | 1. Takes things literally; e.g. does not understand certain expressions.  2. Does not understand jokes.  3. Does not fully understand the content of conversations.  4. Is exceptionally naive; believes anything you say.  5. Frequently says things that are not relevant to the conversation.  6. Talks confusedly; jumps from one subject to another when talking.  7. Only talks about things that are of concern to himself/herself. |
| ***Stereotypy*** | ***Resistance*** |
| 1. Constantly touches objects.  2. Smells objects.  3. Makes odd, fast movements with fingers or hands.  4. Is extremely pleased by certain movements and keeps doing them.  5. Flaps arms/hands when excited.  6. Is fascinated by certain colors, forms, or moving objects.  7. Sways to and fro.  8. Is unusually sensitive to certain sounds. | 1. Clams up in new situations or when experiencing changes.  2. Panics in new situations or when experiencing changes.  3. Resists changes. |
