# Supplementary material for: Decreased Left Caudate Volume Is Associated with Increased Severity of Autistic-Like Symptoms in a Cohort of ADHD Patients and Their Unaffected Siblings
Source: PLoS One. 2016 Nov 2;11(11):e0165620. doi: 10.1371/journal.pone.0165620 (PMC5091763; doi:10.1371/journal.pone.0165620)
Supplement: S1 Table — (DOCX) [file pone.0165620.s003.docx]

*S3 Table: Distribution of Scanning over Two Sites*

|  | Nijmegen (Avanto) | Amsterdam (Sonata) |
| --- | --- | --- |
| Control | 66 | 88 |
| Unaffected Siblings | 78 | 64 |
| ADHD | 138 | 94 |
